# Universal Screening of Newborns from a Tertiary Care Hospital in South India: A Prospective Analytical Study

**DOI:** 10.1055/s-0045-1809928

**Published:** 2026-03-11

**Authors:** K. Ngohlaki, Kalaiarasi Raja, Akshat Kushwaha, H.T. Lalthanthuami, Lokesh Kumar Penubarthi, Sunil Kumar Saxena, Arun Alexander, Sivaraman Ganesan

**Affiliations:** 1 Department of Otorhinolaryngology, Jawaharlal Institute of Postgraduate Medical Education, Puducherry, India; 2 College of Nursing, Jawaharlal Institute of Postgraduate Medical Education, Puducherry, India

**Keywords:** congenital, hearing loss, newborn infant screenings, brainstem auditory evoked potential, audiologic rehabilitation

## Abstract

**Objective:**

To estimate the prevalence of hearing impairment and significant risk factors for congenital loss in a tertiary hospital in South India.

**Methods:**

Over 3 years, a prospective analytical study was conducted in the Department of Otorhinolaryngology in a tertiary hospital in South India. All newborns born in the hospital or those referred were initially screened using the Transient Evoked Otoacoustic Emissions (TEOAE) in a soundproof room by a trained audiologist at 24 to 48 hours of birth. Results were either “PASS” or “REFER.” Those who did not pass the initial screening were retested with TEOAE at their 6-week vaccination appointment. If the second result was “REFER,” the neonate underwent Brain Evoked Response Auditory (BERA) for confirmation.

**Results:**

A total of 3,679 neonates (1,931 males, 1,748 females) underwent TEOAE testing. Of those, 2,426 (65.9%) passed the first test, and 1,253 (34.1%) were referred. The second TEOAE test was done for 1,174 patients, with results of 1,013 (86.29%) as “PASS” and 161 (13.71%) as “REFER.” The BERA results of 95 neonates showed normal in 84 (88.42%), and 11 (11.58%) had profound hearing loss. The prevalence of hearing impairment in the present study was 0.29% (95% CI: 0.2–0.6%). Major risk factors such as maternal infections, like toxoplasmosis, others (syphilis, hepatitis B), rubella, cytomegalovirus, and herpes simplex (TORCH), craniofacial anomalies, low birth weight < 1,500 g, low Appearance, Pulse, Grimace, Activity and Respiration (APGAR) scores (0–4 at 1 minute and 0–6 at 5 minutes), and mechanical ventilation lasting for 5 days or longer, were significantly associated with hearing impairment at *p* < 0.05.

**Conclusion:**

Newborn hearing screenings must be made mandatory, irrespective of risk factors. This way, early diagnosis and necessary interventions can be implemented.

## Introduction

Nearly 32 million of the 360 million people with debilitating hearing loss are children, constituting around 5% of the world's population. 1 Preventive measures are estimated to potentially reduce childhood hearing loss by over 60%. 1 It is crucial to take proactive measures to prevent avoidable causes of hearing loss and provide the necessary support, including rehabilitation, education, and empowerment, enabling them to achieve their maximum potential. The impact of hearing loss on a child's overall growth depends on factors like the degree of loss, as well as age of onset, identification and intervention. Since universal hearing screening has been implemented, many children with hearing loss can get appropriate help and treatment early. Moreover, due to technological advancements, there are various aid options, including hearing aids or cochlear implants.

In India, approximately 5.82 per 100,000 people have congenital hearing loss. This translates to two children born with deafness every hour, with 18,000 new cases annually. About 5% of the Indian population has speech and hearing problems due to congenital sensorineural hearing loss (SNHL) with delayed speech and language development. Thus, at least 10,000 children with genetically profound hearing loss are added to the current population.

The objective of the present study was to estimate the prevalence of hearing impairment in neonates in a tertiary health care center in South India and to investigate the relationship between hearing status in newborns and various risk factors like family history of hearing loss, maternal infections like toxoplasmosis, others (syphilis, hepatitis B), rubella, cytomegalovirus, and herpes simplex (TORCH), craniofacial anomalies, birth weight < 1,500 g, bilirubin > 20 mg/dl, ototoxic medications taken by the mother, bacterial meningitis, appearance, pulse, grimace, activity, and respiration (APGAR) scores of 0 to 4 at 1 minute and 0 to 6 at 5 minutes, mechanical ventilation lasting for 5 days or longer, syndromes associated with SNHL or conductive hearing loss, and preterm deliveries.

## Methods

A prospective analytical study was conducted in the Department of Otorhinolaryngology in a tertiary health care center in South India over 3 years. The Institute's Ethics Committee JIP/IEC/2021/173 approved the study. All newborns delivered in tertiary health care centers or referred for screening underwent hearing screening using the Transient Evoked Otoacoustic Emissions (TEOAE) between 24 and 48 hours after birth. After obtaining the parents' consent, detailed histories were taken, including high-risk factors like family history of hearing loss, maternal infections like TORCH, craniofacial anomalies, birth weight less than 1,500 g, hyperbilirubinemia requiring exchange transfusion, ototoxic medications used by the mother etc. Mothers of all babies received counselling regarding the benefits of hearing screening, the procedure, the need for follow-up, and further tests after failed screening.

The first screening test occurred in a soundproof room, and a trained audiologist conducted the TEOAE test. Results were either “PASS” or “REFER.” Parents of babies who did not pass the screening test were offered counselling, and a repeat TEOAE screening was conducted at 6 weeks of age during their vaccination visit after ear examination to rule out ear wax/debris. The second screening was performed in the soundproof room. Babies who passed the second screening were discharged from the study, while those referred were scheduled for Brain Evoked Response Auditory (BERA) testing. The final statistical analysis was done using the IBM SPSS Statistics for Windows (IBM Corp.), version 25.0.

## Results

**Fig. 1:**
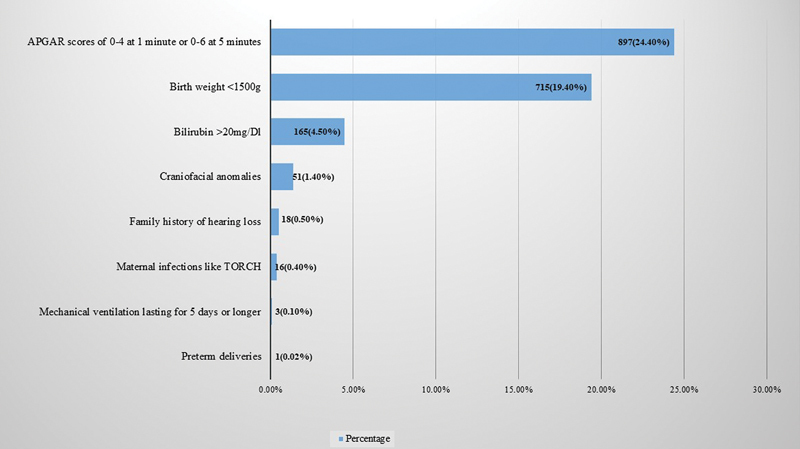
Distribution of major risk factors among the study population.

The present study screened 3,679 neonates born at or referred to a tertiary hospital for hearing assessment, including 1,931 males (52.5%) and 1,748 females (47.5%). Among the 3,679 screened neonates, major risk factors were present in 1,370 (37.2%; 95% confidence interval [CI]: 35.7–38.8) and absent in 2,309 (62.80%; 95% CI: 61.2–64.3). Among the significant risk factors, the most common was low APGAR scores of 0 to 4 at 1 minute and 0 to 6 at 5 minutes, present in 897 (24.4%) neonates, followed by low birth weight (< 1.5 kg), present in 715 (19.4%), as shown in Figure 1 .

Among the 3,679 subjects who underwent initial hearing screening, 2,426 (65.9%) passed the test, indicating normal hearing, while 1,253 (34.1%) were referred, suggesting possible hearing impairment. Neonates who were ‘REFER’ after the first TEOAE screening underwent a second one. However, 79 subjects could not undergo the second test for various reasons. In total, 1,174 subjects underwent the second hearing test, of which 1,013 (86.29%) passed and 161 (13.71%) were ‘REFER’.

**Fig. 2:**
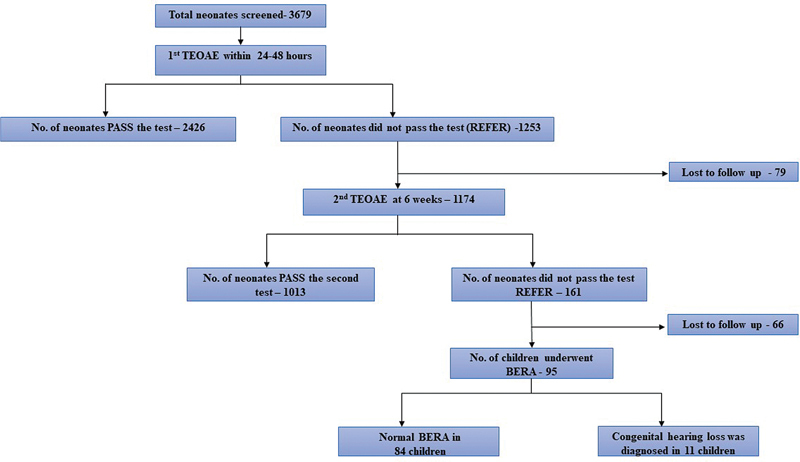
Study results of congenital hearing loss identification through universal newborn screening.

Out of 161 subjects who were ‘REFER’ after the second screening test, 66 were unable to come for follow-up, so BERA was done in 95 subjects, out of which 84 (88.42%) had normal and 11 (11.58%) had abnormal results. The prevalence of hearing impairment in the present study was 0.29% (95%CI: 0.2–0.6%), calculated by considering 11 subjects as hearing impaired out of a total of 3,679 subjects ( Figure 2 ). Major risk factors, such as maternal infections like TORCH, craniofacial anomalies, low birth weight (< 1,500 g), low APGAR scores (0 to 4 at 1 min; and 0 to 6 at 5 min), and mechanical ventilation lasting for 5 days or longer, were significantly associated with hearing impairment ( *p* < 0.05), as shown in Table 1 .

**Table 1:** Association between hearing status and major risk factors among participants

S. No.	Major risk factors		Hearing status		*p* -value
			Impaired n (%)	Normal n (%)	
1	Family history of hearing loss ^a^	Present	0	18 (100)	0.816
		Absent	11 (0.3)	3,650 (99.7)	
2	Maternal infections like TORCH ^a^	Present	1 (6.3)	15 (93.8)	**< 0.001**
		Absent	10 (0.3)	3,653 (99.7)	
3	Craniofacial anomalies ^a^	Present	3 (5.9)	48 (94.1)	**< 0.001**
		Absent	8 (0.2)	3,620 (99.8)	
4	Birth weight < 1,500 g ^a^	Present	6 (0.8)	709 (99.2)	**0.003**
		Absent	5 (0.2)	2,959 (99.8)	
5	Bilirubin > 20 mg/dl ^a^	Present	0	165 (100)	0.472
		Absent	11 (0.3)	3,503 (99.7)	
6	APGAR scores (0–4 at 1 min; or 0–6 at 5 min) ^a^	Present	6 (0.7)	890 (99.3)	**0.019**
		Absent	5 (0.2)	2,778 (99.8)	
7	Mechanical ventilation ≥5 days ^b^	Present	1 (25.0)	3 (75.0)	**0.012**
		Absent	10 (0.3)	3,665 (99.7)	
8	Preterm delivery ^b^	Present	0	1 (100)	0.956
		Absent	11 (0.3)	3,667 (99.7)	

**Abbreviations:** %, percentage; APGAR, appearance, pulse, grimace, activity, respiration; n, number of participants; TORCH, toxoplasmosis, rubella, cytomegalovirus, herpes simplex.

**Notes:**^a^ Chi-square test. ^b^ Fisher's exact test; *p* < 0.05.

Multiple logistic regression was performed to analyze the association between hearing impairment and five major risk factors identified in the present study ( Table 2 ).

**Table 2:** Multiple logistic regression for predictors of hearing impairment

S. Number	Major risk factors		Hearing status		AOR (95%CI)	*p* -value
			Impaired n (%)	Normal n (%)		
1	Maternal infections like TORCH ^a^	Present	1 (6.3)	15 (93.8)	43.75 (4.67–409.39)	0.001
		Absent	10 (0.3)	3,653 (99.7)	Ref	
2	Craniofacial anomalies ^a^	Present	3 (5.9)	48 (94.1)	53.92 (11.96–243.12)	< 0.001
		Absent	8 (0.2)	3,620 (99.8)	ref	
3	Birth weight < 1,500 g ^a^	Present	6 (0.8)	709 (99.2)	4.34 (1.09–17.26)	0.037
		Absent	5 (0.2)	2,959 (99.8)	ref	
4	APGAR (0–4 at 1 min; 0–6 at 5 min) ^a^	Present	6 (0.7)	890 (99.3)	2.43 (0.66–8.99)	0.184
		Absent	5 (0.2)	2,778 (99.8)	ref	
5	Mechanical ventilation ≥5 days ^b^	Present	1 (25.0)	3 (75.0)	114.23 (8.72–1,496.99)	< 0.001
		Absent	10 (0.3)	3,665 (99.7)	ref	

**Abbreviations:** AOR, adjusted odds ratio; TORCH, toxoplasmosis, rubella, cytomegalovirus, herpes simplex; APGAR, appearance, pulse, grimace, activity, respiration; CI, confidence interval; n, number of participants.

**Notes:**^a^ Chi-square test. ^b^ Fisher's exact test; *p* < 0.05.

Neonates with maternal infections like TORCH have a 43.75 times higher risk of having hearing impairment as compared with neonates without TORCH infections after adjusting the effect of other major risk factors (Craniofacial anomalies, birth weight <1,500 g, APGAR scores of 0 to 4 at 1 minute and 0 to 6 at 5 minute, and mechanical ventilation lasting for ≥ 5 days). Neonates with craniofacial anomalies have a 53.92 times higher risk of hearing impairment than those without, after adjusting for other major risk factors. Also, neonates with birth weights of less than 1,500 g have a 4.34 times higher risk of hearing impairment than those without. Neonates with a history of mechanical ventilation lasting for 5 or more days have a 114.23 times higher risk of hearing impairment than neonates without it, after adjusting the effect of other major risk factors. Neonates with low APGAR scores have a 2.43 times higher risk of having hearing impairment as compared with those without. However, there is a 95% probability that the true odds ratio (OR) of the population lies between 0.66 and 8.99, which is not statistically significant ( *p* = -0.184).

## Discussion

Screening programs that solely target infants with one or more risk factors for hearing loss typically only cover 50% of children with this impairment. 2 Consequently, children with known risk factors for hearing loss are often identified at an average age of 11 to 19 months, while those without apparent risk are identified at 15 to 19 months. 3 4 Remarkably, around 50% of newborns with permanent bilateral congenital hearing loss do not exhibit identifiable risk indicators. This highlights it is critical to screen all newborns, regardless of risk factors. This becomes particularly significant in countries like India, where 5% of the population suffers from speech and hearing impairment due to congenital conditions.

In the current study, out of a total of 3,679 newborns who were screened, hearing loss was present in 11, making the prevalence of hearing impairment 0.29%. A study by Gouri et al., 5 conducted at a tertiary care hospital in Rajasthan, found that the incidence of hearing impairment was 0.23%, comparable to the present study. Additionally, the study identified low APGAR scores (< 5 at 5 min), Neonatal Intensive Care Unit (ICU) admission, fetal distress, and meconium-stained amniotic fluid as significant predictors of congenital hearing loss. This is again comparable to the present study, due to having subjects from the same country and similar predisposing factors.

John et al. studied 500 newborns and found a 0.6% prevalence of hearing impairment. 6 This difference compared with the present study might be because of the smaller sample size, with most of them having risk factors. In comparison, the present study included more newborns from a universal screening, disregarding risk factors.

Nagapoornima et al. conducted a study on 1,769 neonates, finding that those with a family history of hearing loss had the highest incidence of impairment. 7 However, in the present study, 18 patients with normal hearing had a family history of the condition. This highlights the need to perform screening on all newborns, irrespective of their risk factors.

Besen et al. investigated the association between hearing impairment and congenital syphilis. Among 21,434 evaluated newborns, 1.6% had hearing impairment, and 1.7% had congenital syphilis. The findings suggested that neonates with congenital syphilis were 3.25 times more likely to fail the universal screening. 8 Consistent with previous research, TORCH infection was significantly linked to this condition. Even though some babies without congenital syphilis also had hearing loss, these findings also underscore the importance of the universal newborn hearing screening.

Barreira-Nielsen et al. 9 investigated progressive hearing loss in 330 newborns and found that about half of the babies had worsening while the other half remained stable. Intriguingly, this study found an inverse association with craniofacial anomalies, contrasting the present study findings, which revealed a 53.92 times higher risk among newborns with craniofacial anomalies than those without after controlling for other significant risk factors. This discrepancy may be attributed to the smaller sample size in their study. Another study, by Sheapp et al., confirmed this association. 10

Driscoll et al. 11 studied the association between family history and pediatric hearing loss, recruiting 380,895 children. The yield of congenital hearing loss in children with family history as their sole risk factor was 1.43%. Those with the presence of family history who passed screening tests were followed up for 6 months, or until they reached 3 years of age, and the yield of postnatal hearing loss in these children was found to be 1.7%. 11 This calls for continuing hearing screening at time intervals to detect hearing impairment at the early stage.

Pruszewicz et al. studied the relationship between low birth weight and hearing impairment. Although very low birth weight did not independently predict this condition, a cluster of risk factors, including it, was significantly associated with hearing impairment. 12 In the present study, newborns with very low birth weight were found to have a 4.34 times higher risk of hearing impairment than those without. This discrepancy might be attributed to the larger sample size in the present study, compared with the 100 children in their research.

A review of hearing loss in children with very low birth weight by Cristobal et al. showed that the survival rate of this cohort in ICUs had increased and that this variable was commonly associated with multiple other risk factors that could alter hearing synergistically. Hearing loss in the early years of life can lead to pathology in auditory processing. Therefore, long-term follow-up of babies with very low birth weight and other risk factors was declared a necessity. 13

Teixeira et al. 14 studied the impact of hyperbilirubinemia on newborn hearing. A review of seven papers was done, and it was found to be a significant association. The results were similar to Peyvandi et al. 15 Auditory changes from bilirubin toxicity were found to be reversible before 2 years of life in one study, while in another, it remained even after phototherapy or exchange transfusion, indicating the need for audiological follow-up.

Kvestad et al. 16 studied the relationship between sensorineural hearing loss in children and APGAR score. It was a registry-based study conducted in Norway, recruiting 3,92,371 children. It was found that low scores of < 3 were significantly associated with SNHL. 16 . In the present study, low APGAR was significant but insignificant when considering other risk factors. Da Silva et al. studied their associations on the first hearing screening of neonates. 17 Our results showed that low APGAR scores also increased the chances of REFER in the TEOAE in newborns above 1,500 g without peri-intraventricular hemorrhage. However, other risk factors were also present in newborns with this test.

Rastogi et al. studied the effects of ventilation on hearing loss in neonates and found an association. 18 This holds in the present study, where newborns requiring mechanical ventilation for 5 days or longer had 114.23 times the risk of hearing impairment compared with those who did not.

Zhu et al. performed a study of preterm infants and found that preterm infants have a higher risk of hearing impairment due to their fragile auditory nervous systems. 19 However, in the present study, we found no significant association, possibly due to an increased number of other risk factors.

Ching et al. 20 studied the effect of age on intervention for permanent hearing loss and found that early intervention was vital for mitigating language deficits in children with hearing loss. Earlier amplification and cochlear implantation significantly improved language outcomes. Children receiving interventions at 24 months had poorer language skills than those starting at 3 months. 20 While the present study didn't find direct impacts of screening on the outcomes, early identification remains crucial for timely intervention.

Ezzeldin et al. 21 conducted a neonatal hearing screening on 60 newborns, dividing them into hyperbilirubinemia ( *n* = 30) and control ( *n* = 30) groups. Their study's use of BERA revealed a significant difference in hearing between the two groups. However, TEOAE screenings indicated a higher false-positive rate. Despite this, the ease of administration makes it a complementary tool to BERA. 21 In contrast, in the present study, no statistically significant correlation was found between hearing impairment and bilirubin levels exceeding 20 mg/dL.

Strengths of our study included adequate sample size and in-depth analysis of risk factors and outcomes. Limitations of the study include a significant number of newborns who did not return for confirmatory BERA tests or subsequent evaluations, leading to incomplete data, and a study population that might not be representative of the entire newborn population in South India due to our tertiary care setting, where more complex cases are the norm.

## Conclusion

A two-tier screening system is beneficial, as it screens all newborns regardless of risk factors. This approach is practical since only those who fail the initial or repeat screenings require the more complex and time-consuming diagnostic BERA test. In the present study, the prevalence of hearing impairment in the tertiary care hospital located in South India is 0.29%. The present study identified several risk factors linked to hearing impairment, including maternal infections with TORCH, craniofacial abnormalities, low birth weight (< 1.5 kg), and prolonged mechanical ventilation (≥ 5 days). Our findings reinforce the strong association between these factors and the development of hearing loss.
